# Ellagitannin Content in Extracts of the Chestnut Wood *Aesculus*

**DOI:** 10.3390/molecules29174015

**Published:** 2024-08-25

**Authors:** Taja Žitek Makoter, Maša Knez Marevci, Željko Knez

**Affiliations:** 1 Laboratory for Separation Processes and Product Design, Faculty of Chemistry and Chemical Engineering, University of Maribor, SI-2000 Maribor, Slovenia; taja.zitek@um.si (T.Ž.M.); masa.knez@um.si (M.K.M.); 2 Faculty of Medicine, University of Maribor, Taborska 8, SI-2000 Maribor, Slovenia

**Keywords:** antioxidant activity, chestnut wood, conventional extraction, extraction, fractionation, polyphenols, supercritical extraction, tannins, ultrasound extraction

## Abstract

The chestnut tree (*Castanea sativa* Mill.) is a widespread plant in Europe, rich in high-value compounds, which can be divided mainly into monomeric polyphenols and tannins. These compounds exhibit various biological activities, such as antioxidant, as well as anticarcinogenic and antimicrobial properties. Chestnut wood (CW) extracts were prepared using different extraction techniques, process conditions, solvents, and their mixtures. This work aimed to test various extraction techniques and determine the optimal solvent for isolating enriched fractions of vescalagin, castalagin, vescalin, and castalin from CW residues. Supercritical CO_2_ extraction with a more polar cosolvent was applied at different pressures, which influenced solvent density. According to the results, the proportions of the components strongly depended on the solvent system used for the extraction. In addition, HPLC-DAD was used for semiqualitative purposes to detect vescalagin, castalagin, vescalin, and castalin. The developed valorization protocol allows efficient fractionation and recovery of the polyphenolic components of CW through a sustainable approach that also evaluates pre-industrial scaling-up.

## 1. Introduction

**Figure 1:**
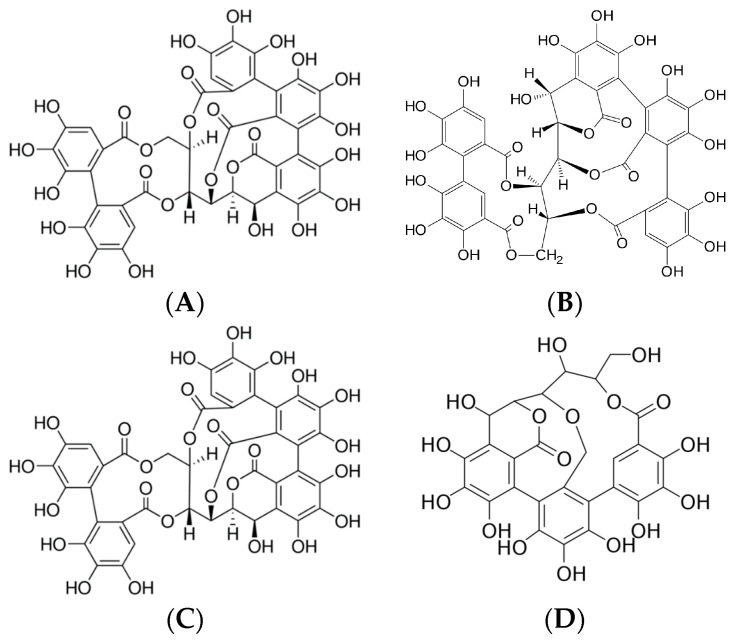
(**A**) Vascalagin (C_41_H_26_O_26_), (**B**) castalagin (C_41_H_26_O_26_), (**C**) vescalin (C_27_H_20_O_18_), (**D**) castalin (C_27_H_20_O_18_).

Since the chestnut tree is a source of several phenolic compounds, especially tannins, *Castanea sativa* Mill. is one of the most important plants used in the tanning industry. Its bark and wood chips are typically used to extract tannins; no other plant parts are used. The bark contains approximately 60% active tanning substances, composed mainly of castalagin, vescalagin, castalin, and vescalin [1]. These plant defense substances, which exhibit a strong astringent taste and precipitate proteins, are divided into two major groups: condensed and hydrolyzable tannins. Condensed tannins, also known as proanthocyanidins, are oligomers or polymers composed of flavonoid units without sugar residues [2]. Catechin and epicatechin are the most representative monomeric units in natural condensed tannins, together with epicatechin gallate and, to a lesser extent, gallocatechin, epigallocatechin, afzelechin, and epiafzelechin. Hydrolyzable tannins are composed of esters of gallic acid or ellagic acid with a sugar core, mainly glucose, and are thus divided into two subclasses: gallotannins and ellagitannins, respectively. Indeed, the characteristic compounds of the sweet chestnut are ellagitannins, specifically vescalagin, castalagin, vescalin, and castalin (Figure 1) [3].

They showed that chestnut shell extracts possess higher antioxidant activity and a greater amount of phenolics than eucalyptus bark. Vázquez et al. [4] used different extraction solvents for treating chestnut shells, and the highest yield of extract was obtained using water (H_2_O) as a medium (12.2%). The extraction yield was improved when 2.5% Na_2_SO_3_ was added to the water (yielding 25.62%). Their following study [5] showed that the extraction of chestnut shells gave the highest yield of 49.4% if 10% NaOH was added to the water. Vasconcelos et al. [6] used water (H_2_O), 70% methanol (MeOH), 70% ethanol (EtOH), 70% acetone (Ac), and methylethylketone as extraction solvents for four Portuguese chestnut shell cultivars. The highest yield of total phenols, total condensed tannins, and low-molecular-weight phenolics was obtained using 70% acetone at 20 °C. However, investigations dealing with the hydrothermal treatment of chestnut as an eco-friendly method are still scarce in the literature. Moure et al. [7] investigated the hydrolytic treatment of chestnut burs, and it was shown that extracts were produced with good bioactive properties. Furthermore, data on the optimization of the extraction process of chestnut are limited in the literature. Reinoso et al. [8] studied the optimization of antioxidants obtained by the extraction of chestnut leaves using 96% ethanol, methanol, and acidified water as extraction solvents, while Aires et al. [9] described the extraction and optimization of polyphenols, tannins, and ellagitannins obtained from chestnut peels using water, Na_2_SO_3_, and NaOH at different concentrations. The extracts are complex mixtures of several substances. Among them are a suitable quantity of organic acidic compounds, which determine their considerable astringency and capability to be mixed with other agents in the tanning industry. However, scarce information is available about the potential use of other types of chestnut wastes, e.g., chestnut peels. Although several studies have indicated that chestnuts are a rich source of tannins [10], the majority of research studies evaluate the use of leaves, galls, bark, and wood [9], and only a few are devoted to the study of the potential use of skins and peels from the nuts. The present study outlines the entire extract preparation procedure using different types of solvents at varying solvent grades and extraction procedures. Finally, the optimal conditions were determined for obtaining an extract with the highest possible content of vescalagin, castalagin, vescalin, and castalin. The content of the condensed tannins of cultivars of CW after extraction was analyzed using HPLC-DAD in order to evaluate the hypothesis that chestnut wastes could be used as an antioxidant with antimicrobial activity.

## 2. Results and Discussion

### 2.1. Recovery of Ellagitannins Extracted Using Different Extraction Techniques

The extraction efficiency of ellagitannins from wood varied across different solvents, process conditions, and extraction methodologies. The extracts analyzed in this study demonstrated a high content of phenolic compounds, with tannins such as vescalagin and castalagin being particularly prominent. Ultrasound-assisted extraction is a quicker procedure, which is economically more feasible due to lower solvent consumption and lower extraction temperatures, and therefore, has a lower impact on the environment and the final product. Additionally, water is the cheapest and most environmentally benign solvent [11], with the further advantage of the ability to extract polysaccharides, which may have beneficial effects in synergy with ellagitannins [12,13,14,15]. Results on recovery of ellagitannins extracted using different extraction methods is given in Appendix A.

**Figure 2:**
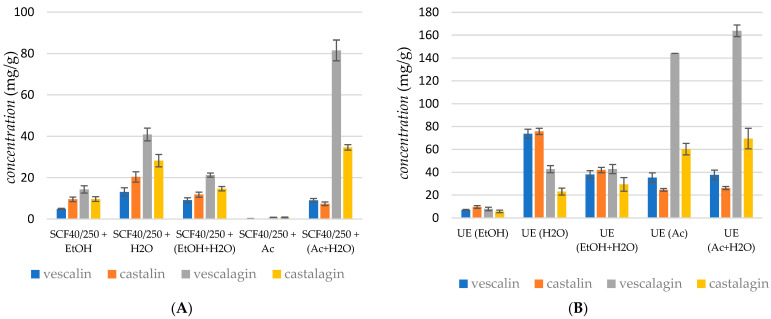

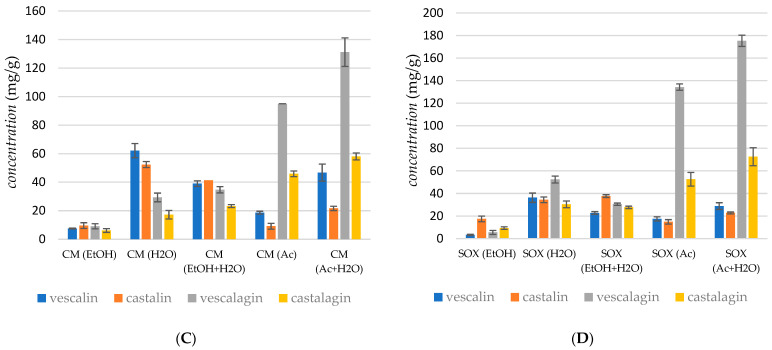
Recovery of ellagitannins extracted using (**A**) supercritical fluid extraction, (**B**) ultrasound extraction, (**C**) cold maceration, and (**D**) Soxhlet extraction. Error bars represent standard deviations.

Figure 2A represents the obtained yields of vescalagin, castalagin, vescalin, and castalin in the case of supercritical CO_2_ extraction at a pressure of 250 bar and a temperature of 40 °C using five different cosolvent systems: EtOH, H_2_O, EtOH + H_2_O, Ac, and an Ac + H_2_O mixture.

The Shapiro–Wilk test indicated that the sum of tannin concentrations in the extract is normally distributed (*p* = 0.1218), which justifies the use of Analysis of Variance (ANOVA). ANOVA revealed significant differences among the data (*p* < 0.0005). Furthermore, the post hoc Tukey test identified significant differences between individual conditions, including between the two most productive: Ac + H_2_O and H_2_O (*p* < 0.0005). It can be noticed that yield depended on both the extraction solvent and the type of extraction method. In the case of supercritical fluid extraction, the presence of an entrainer and the modified polarity of the extraction media represent key parameters in phenolic recovery. The experimental results showed that the yield of ellagitannins increased with the polarity of the solvent; the highest yield of vescalin (9.06 mg/g) was obtained using the Ac + H_2_O mixture to modify the low polarity of supercritical CO_2_. The presence of H_2_O in the extraction media was proved to be convenient for extracting vescalagin, whilst the lowest recovery of vescalagin was attained in the case of supercritical CO_2_ extraction using Ac as the cosolvent (2.2 mg/g). The highest recovery of castalagin was obtained with the Ac + H_2_O mixture as the cosolvent (34.66 mg/g). It was observed that, in the case of supercritical CO_2_ extraction, the content of ellagitannins was highest when the polarity of the solvent was modified with H_2_O, followed by Ac and EtOH + H_2_O.

Škerget and co-workers reported on the influence of temperature. Namely, the degradation rate of ellagic acid is probably slower than the rate of its production through the hydrolysis of ellagitannins. Vescalin and castalin were present in trace amounts at the low temperature of 150 °C. Gallic acid and ellagitannins were no longer present in the samples above 200 °C. Furthermore, a solvent–solid ratio of 30 mL/g resulted in higher yields of almost all these compounds compared to a ratio of 10 mL/g. Generally, the experimental results showed that the yield of ellagitannins decreased when the temperature increased [16]. This study also reported that ellagitannins were not stable and were hydrolyzed into ellagic acid at high temperatures under subcritical conditions. In our work, supercritical CO_2_ extraction using H_2_O as the cosolvent was performed at a temperature of 40 °C. The recovery of tannins was relatively low in supercritical media; a somewhat higher yield of vescalagin and castalagin was observed when a mixture of Ac Ac + H_2_O was applied as the cosolvent. This was expected since Ac and the mixture of Ac + H_2_O provided the highest recovery yields independently of the extraction technique.

Figure 2B represents the obtained yields of vescalagin, castalagin, vescalin, and castalin in the case of ultrasonic extraction at a temperature of 40 °C using five different solvent systems: EtOH, H_2_O, EtOH + H_2_O, Ac, and the Ac + H_2_O mixture. The Shapiro–Wilk test indicated that the sum of tannin concentrations in the extract was not normally distributed (*p* = 0.03879), necessitating the use of the Kruskal–Wallis test. The Kruskal–Wallis test revealed significant differences among the data (*p* = 0.00974). Additionally, the post hoc Dunn test identified significant differences between certain conditions, including between the two most productive: Ac + H_2_O and Ac (*p* = 0.0214).

Recovery of compounds depended on the polarity of the extraction solvent. Again, vescalagin was the most extractable tannin; the highest yields of vescalagin (163.85 mg/g) were obtained using the Ac + H_2_O mixture and using Ac as the cosolvent (144.13 mg/g). The highest recovery of castalagin was obtained in the presence of Ac, whereas the extraction yields of other compounds were less dependent on the extraction medium. It was observed that, in the case of ultrasonic extraction, the content of ellagitannins was the highest when the polarity of the solvent was modified with Ac, followed by the Ac + H_2_O mixture. The total recovery in ultrasonic extraction was higher compared to supercritical extraction.

Since the specific structure and reactions of ellagitannins are related to their solubility, which is related to the complex extraction procedure, our work comprises variations in extraction procedures, process conditions, and extraction media. According to the literature, different solvents have been used to obtain chestnut extracts, including methanol and trifluoroacetic acid, as catalysts for the subsequent hydrolysis of the extract. The results show that hydrolyzed chestnut bark contains a considerably higher amount of ellagic acid compared to unhydrolyzed bark. Comandini et al. [1] performed the extraction of chestnut bark in methanol for 30 min at room temperature and then sonicated the sample in a water bath. Živković et al. [14] performed the extraction of different parts of sweet chestnuts (leaves, catkins, seed, bark, and burs) via ultrasound using 50% ethanol. Reddy et al. [15] extracted chestnut bark with methanol in order to study the cardiovascular effects of the extracts. Besides ellagic acid and ellagitannins, chestnut trees also contain gallic acid, which is one of the main compounds in their structure. Chestnut processing generates waste products, which mainly include shells, skins, and burs. However, Vázquez et al. [4] investigated the antioxidant activity and chemical composition of chestnut shells and eucalyptus bark.

Figure 2C represents the yields of vescalagin, castalagin, vescalin, and castalin obtained in cold maceration. The Shapiro–Wilk test indicated that the sum of tannin concentrations in the extract is normally distributed (*p* = 0.0518), supporting the use of Analysis of Variance (ANOVA). ANOVA revealed significant differences among the data (*p* < 0.0005). The post hoc Tukey test further demonstrated significant differences across most conditions, including between the two most productive solvents: Ac + H_2_O and H_2_O (*p* < 0.0005). However, no significant difference was observed between the solvents H_2_O and Ac (*p* = 0.3957). The recovery of vescalagin depends on the polarity of the extraction solvent; the highest yield of vescalagin (131.57 mg/g) was obtained using the Ac + H_2_O mixture and using Ac as the solvent (95.04 mg/g). A similar trend was observed for castalagin. Figure 2D represents the obtained yields of vescalagin, castalagin, vescalin, and castalin in the case of SOX extraction. The Shapiro–Wilk test indicated that the sum of tannin concentrations in the extract is normally distributed (*p* = 0.2218), supporting the use of Analysis of Variance (ANOVA). ANOVA revealed significant differences among the data (*p* < 0.0005). The post hoc Tukey test further demonstrated significant differences between individual conditions, including between the two most productive solvents: Ac + H_2_O and Ac (*p* < 0.0005). The recovery of vescalagin depends on the polarity of the extraction solvent; the highest yield of vescalagin (175.35 mg/g) was obtained using the Ac + H_2_O mixture. The extraction with Ac gave a somewhat lower yield of vescalagin, about 134.30 mg/g.

The concentration of tannins and other phenols in the chestnut bark samples analyzed varied widely, ranging from 0.02 to 18.38 mg/g, which is comparable to the results reported by Comandini et al. [1]. The different amounts, as well as the qualitative composition of tannins detected in the commercial chestnut bark samples, might be due to a different phenolic profile of the raw materials used in the manufacturing process or to losses that occurred during the different manufacturing processes. Moreover, the different physical states of the chestnut bark samples analyzed (powder, granular, and coated) might have influenced the global amount of tannin extracted and could have led to preferential extraction of some classes of phenols. An important conclusion is that the yield of ellagitannins, particularly vescalagin and castalagin, was influenced by the polarity of the extraction solvent. 

According to their results, it can be concluded that the extraction method and processing conditions influenced the amounts of extracted tannins. Overall, supercritical CO_2_ extraction with Ac + H_2_O mixtures yielded high concentrations of vescalagin (81.47 mg/g), whereas ultrasonic extraction with Ac alone achieved the highest overall recovery of vescalagin (163.85 mg/g). In contrast, cold maceration and Soxhlet extraction also produced substantial yields, with the Ac + H_2_O mixture proving particularly effective in the latter. Further analysis indicated that extraction methods and conditions affected the yield of ellagitannins, with higher temperatures generally leading to reduced yields. Supercritical CO_2_ extraction using H_2_O as a cosolvent at 40 °C yielded relatively low amounts of tannins, suggesting that using Ac and Ac + H_2_O mixtures as cosolvents was more effective across different extraction techniques.

The concentration of tannins and other phenolic compounds in the chestnut bark samples ranged from 0.2 to 183.8 mg/g, aligning with the literature values [10] and highlighting the variability in tannin content due to differences in raw materials and extraction methods. These findings underscore the importance of solvent choice and extraction conditions in maximizing the yield and stability of ellagitannins.

### 2.2. Effect of the Extract on WM-266-4 Cells

**Figure 3:**
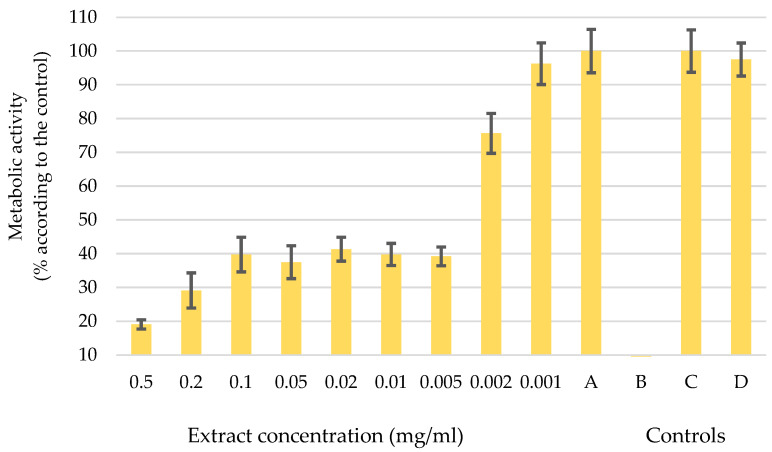
Metabolic activity of melanoma cells after the application of a water-based UE extract at different concentrations (0.5 mg/mL–0.001 mg/mL) and controls: A: melanoma cells WM-266-4 and medium only, B: medium and extract, C: healthy skin cells NHME, and D: NHEM cells and extract. Error bars represent standard deviations.

In pursuit of environmentally conscious methods and recognizing the importance of sustainable and cost-effective practices, it was decided to advance our research efforts with an extract obtained through ultrasonic extraction using H_2_O as the solvent. The diagram in Figure 3 shows the metabolic activity of melanoma cells WM-266-4 after the application of the water-based UE extract in different concentrations from 0.5 mg/mL to 0.001 mg/mL. The letters A, B, C, and D show the controls used to verify the accuracy of the data. The diagram shows that concentrations up to 0.002 mg/mL influence the change in metabolic activity of melanoma cells. For the concentration of 0.001 mg/mL, the error bars are in the control range, which means that such a concentration has no influence on the function of the cells.

A decrease in metabolic activity was observed upon the application of a concentration of 0.005 mg/mL to WM-266-4 cells. The melanoma cells showed a considerable reduction in their metabolic activity, which dropped to 40% of its initial level. When the concentration increased to 0.2 mg/mL and 0.5 mg/mL, the metabolic activity further dropped to 30% and 20%, respectively. However, the effects of such high concentrations should be considered carefully, as it is plausible that the sheer amount of the extract itself exerted an influence and not just its inherent activity. However, the concentration of 0.005 mg/mL was defined as the optimal dose of extract administered to melanoma cells, a determination that is supported by the results of the controlled experiments. In Control A, the melanoma cells remained fully functional, which was consistent with our predictions. Control B, on the other hand, showed an absence of organisms, confirming the purity of our extract without any contamination. The additional Controls C and D confirmed the authenticity of the results, as normal human epidermal melanocytes (NHEM) showed uninterrupted growth and division. It is noteworthy that the chosen dosage of the extract did not appear to have any discernible effect on healthy cells (control D). These results are important indicators for subsequent investigations into the potential therapeutic effects of the extract as a complementary therapy in cancer treatment.

Water bark extracts contain a variety of bioactive compounds, including polyphenols, alkaloids, flavonoids, lignans, and terpenes, which have significant anticancer potential. These compounds have an antioxidant effect, inhibit cancer cell-activating proteins, activate DNA repair mechanisms, and stimulate the formation of protective enzymes [17]. Studies have shown that phenolic compounds in bark extracts, such as protocatechuic acid, gallic acid, and catechin, are effective against various cancer cell lines, including breast, cervical, and leukemia cells [18]. In addition, combinations of polyphenols, such as EGCG and quercetin, exhibit synergistic effects that enhance their anticancer properties [19,20,21]. Alkaloids and terpenes from bark extracts also show cytotoxic effects against cancer cells, further emphasizing the potential of these natural extracts in cancer therapy [22,23]. Overall, water bark extracts are a valuable source of compounds with strong anticancer activity, making them promising candidates for further research and possible therapeutic applications [24,25,26]. In our study, water bark UE extracts were tested for antioxidant activity and the effect of the same extract on microorganisms (*S. aureus, E. coli*, and *C. albicans*) was tested. The total phenolic content (TPC) was 73.5 ± 92 mg QE/g bark. Antioxidant activity was evaluated using the ABTS and DPPH assays; according to the ABTS assay, antioxidant activity was 317.3 ± 7.22 mg TE/g bark. The DPPH activity expressed as IC_50_ was 3.1 µg/mL. The effect of the extract on microorganisms (*S. aureus*, *E. coli*, and *C. albicans*) was also assessed.

**Figure 4:**
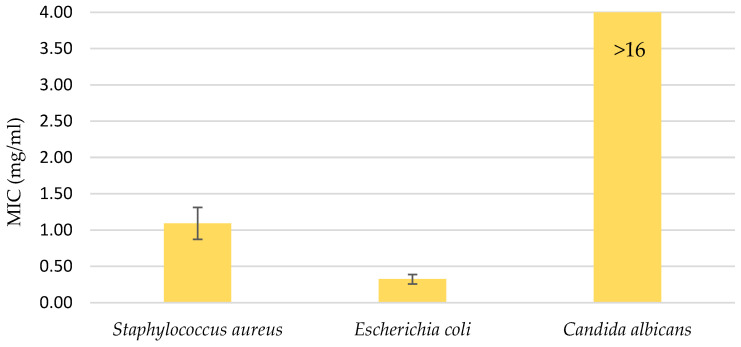
Minimal inhibitory concentration (MIC); antimicrobial activity in the range of water UE extract concentrations from 16 mg/mL to 0.12 mg/mL. Error bars represent standard deviations.

The water UE extract was also applied to three microorganisms: the gram-positive bacteria *Staphylococcus aureus*, the gram-negative bacteria *Escherichia coli*, and the fungi *Candida albicans*. The extract was used in various concentrations, ranging from 16 mg/mL to 0.12 mg/mL. As can be seen from the diagram in Figure 4, the minimum inhibition value for the fungus was not reached, which means that a higher concentration of the extract was required or the extract did not inhibit the effect of *Candida albicans*. The MICs were determined for the two bacteria: 1.092 mg/mL for *S. aureus* and 0.323 mg/mL for *E. coli*.

## 3. Materials and Methods

### 3.1. Plant Material

The chestnut wood bark was delivered from Tanin Sevnica Kemična industrija d.d. and already chopped into small pieces and dried. In addition, the wood was lyophilized to completely remove water from the material. Prior to use, the material was further ground to obtain pieces approximately 1 cm × 1 cm in size.

### 3.2. Chemicals

Acetone (CAS Reg. 67-64-1) and ethanol (CAS Reg. No. 64-17-5) with purity ≥ 99.9 were purchased from Sigma-Aldrich Chemie GmbH (Steinheim, Germany). Carbon dioxide (CAS Reg. No. 124-38-9) with a purity of 99.99% was purchased from MESSER (MG-Ruše, Slovenia). The following standards were used in the HPLC analytical procedure: Vescalagin analytical standard (Sigma-Aldrich Chemie GmbH, Steinheim, Germany, CAS Number: 36001-47-5), Castalagin analytical standard (Sigma-Aldrich Chemie GmbH, Steinheim, Germany, CAS Number: 24312-00-3), Vescalin analytical standard (Sigma-Aldrich Chemie GmbH, Steinheim, Germany, CAS Number: 149-91-7), and Castalin analytical standard (Sigma-Aldrich Chemie GmbH, Steinheim, Germany, CAS Number: 19086-75-0). 

### 3.3. Extractions

The average particle diameter of the material subjected to further extraction was 1.0 cm. The samples were extracted using different extraction methods—ultrasound, Soxhlet, cold, and supercritical fluid. Material from the same batch was applied to all experiments. Furthermore, various solvents and cosolvents were employed: ethanol (EtOH), acetone (Ac), 50% aqueous ethanol (EtOH + H_2_O), 50% aqueous acetone (Ac + H_2_O), and water (H_2_O). Afterward, the obtained extracts were evaporated (BÜCHI Rotavapor R-114 and BÜCHI Vacuum Controller B-721, Uster, Switzerland), and the solvent was removed to dry under reduced pressure. All the obtained extracts were stored at −20 °C until further assays.

The extraction yield (mass of extract/mass of dry material) was used to evaluate the effects of the extraction conditions. The recovery of ellagitannins extracted using different extraction methods is provided as mg of compound per g of extract.

#### 3.3.1. Ultrasound Extraction (UE)

The dried and ground material (20 g) was introduced to an Erlenmeyer flask, and 250 mL of solvent was added, where different solvents and mixtures were used as the extraction media. Then, the Erlenmeyer flask was immersed in an ultrasonic bath (Iskra-Pio, Šentjernej, Slovenia) operating at a fixed power of 40 kHz, with the liquid level in the Erlenmeyer flask kept lower than that of the bath. The extraction was performed at a constant temperature of 40 °C for 1.5 h [27]. 

#### 3.3.2. Soxhlet Extraction (SE)

The Soxhlet extraction was performed using a Soxhlet apparatus ISOLAB NS29-32 (Merck KGaA, Darmstadt, Germany). Twenty grams of dried and ground material was introduced into the tube, and 150 mL of solvent was added to the flask. The extraction was carried out in three cycles for approximately 2 h. The heating temperature was adjusted to the boiling point of the employed solvent [28]. 

#### 3.3.3. Cold Extraction (CE)

The dried and ground material (20 g) and solvent (250 mL) were added to an Erlenmeyer flask. To avoid constant stirring, a magnetic grain was added to the mixture, and the flask was placed on a magnetic stirrer. The extraction took place for about 2 h at room temperature.

#### 3.3.4. Supercritical Fluid Extraction (SFE)

The experiments were performed on a semi-continuous high-pressure flow apparatus designed for a maximum pressure of 500 bar and a temperature of 100 °C. The procedure for the lab-scale extraction process has already been described in previous research [29]. The extractions were carried out in cycles at a pressure of 250 bar and a temperature of 40 °C. Approximately 15 g of dried ground material was charged into the extractor (*V* = 60 mL). The solvent flow rate was kept constant at 1 mL/min, and the solvent-to-feed ratio was 8.2. The water bath temperature was regulated and maintained at a constant level (±0.5 °C, LAUDA DR. R Wobser GmbH & Co. KG, Lauda Königshofen, Germany). The apparatus was first purged with nitrogen and, later, with the gas used for extraction. Next, liquefied gas (CO_2_) was continuously pumped with a high-pressure pump (ISCO syringe pump, model 260D, Lincoln, Nebraska, *P*_max_ = 450 bar) through the preheating coil and over the bed of the sample in the extractor. The solvent flow rate was measured with a flow meter (ELSTER HANDEL GmbH, Mainz, Germany). The product precipitated in a separator (glass trap), where the separation was performed at atmospheric conditions [28].

### 3.4. Determination of Total Phenol Content and Antioxidant Assays

The total phenolic content (TPC) was measured using the Folin–Ciocalteu assay [28]. The extract was mixed with 2.5 mL of 10-fold diluted Folin–Ciocalteu reagent, and then 0.7 M Na_2_CO_3_ was added. The mixture was heated at 50 °C for 5 min, cooled, and the absorbance was measured at 760 nm. The results were expressed as milligrams of quercetin equivalents per gram of dry bark (mg QE/g bark). Antioxidant activity was evaluated using the ABTS and DPPH assays. The ABTS assay was performed according to Stratil et al. (2007) with Trolox as the standard [30]. Absorbance was measured after 10 min at 734 nm, and results were expressed as milligrams of Trolox equivalents per gram of dry bark (mg TE/g bark). The DPPH assay was performed using a modified method by Sharma and Bhat (2009) [31]. The ability of the extract to scavenge DPPH radicals was evaluated by mixing 2090 μL of methanol, 900 μL of methanolic DPPH solution (2 × 10^−4^ M), and 10 μL of the extract, incubating in the dark for 30 min and measuring the absorbance at 515 nm. The results were expressed as IC_50_ (μg/mL), which corresponds to the extract concentration required to inhibit 50% of DPPH radicals.

### 3.5. Identification of Isolated Compounds Using HPLC

An analytical High-Performance Liquid Chromatography (HPLC) system (Vanquish Core, Thermoscientific, Waltham, MA, USA), specifically the Agilent 1100 Series (Agilent Technologies, Waldbronn, Germany), was utilized to analyze the composition of each fraction after each purification and to determine the purity of the final isolates. The column used was an Agilent Eclipse XDB-C18, with 5 µm particles and dimensions of 4.6 mm × 150 mm, maintained at a constant temperature of 40 °C. The solvent system consisted of solvent A [water–formic acid (996:4, *v*/*v*)] and solvent B [methanol–formic acid (996:4, *v*/*v*)]. The elution gradient was adjusted as follows: initially 0% B with a flow rate of 0.55 mL/min for 7.5 min; increased to 0.70 mL/min at 8 min; then gradually to 20% B at 25 min, maintaining the same flow rate. The gradient reached 50% B at 35 min, transitioning to 100% B by 37 min and maintaining this until 45 min. Post-run, the gradient was reset to 0% B with a flow rate of 0.55 mL/min for 10 min. Ellagitannins in the samples were identified by comparing their chromatographic retention times. For the calculation of ellagitannin content, we performed a quantitative analysis based on standard curves generated from known concentrations of pure ellagitannins. 

The standards mentioned in the methods were used during the analytical procedure with the HPLC method. Preliminary, the purity of the standards was verified using MS. 

### 3.6. Antimicrobial and Anticarcinogenic Potential of the Extract

The effect of the extract on WM-266-4 cells and microorganisms (*S. aureus*, *E. coli*, and *C. albicans*) was investigated using the WST-8 assay [32] and the minimum inhibitory concentration (MIC) method. These procedures followed the Standard protocols described by Žitek et al. [29,33]. Briefly, cells and microorganisms were cultured according to known protocols, and then the extract was administered at different concentrations. The WST-8 assay was used to measure the metabolic activity (MA) of the cells after the application of the extract. Data integrity was ensured by including control groups: Control A included cells and medium only, Control B included medium and extract only, Control C included cultured healthy skin cells (normal human epidermal melanocytes), and Control D included NHEM cells and extract. Control A aimed to obtain WM-266-4 cells in the division phase with true forms, while Control B showed no changes. Control C contained normally grown NHEM cells in the division phase and served as a comparative reference for Controls C and D. In the case of microorganisms, three controls were used: the first contained only the medium, the second did not contain the microorganism, and the third did not contain the extract. Results for the cells are given as percentages, indicating the percentage of the metabolic activity of the cells after the application of the extract. Results for microorganisms indicate the minimum inhibitory concentration required to stop the action of the microorganism.

### 3.7. Statistical Evaluation of Results

A study of the statistical data was performed to evaluate the different effects of solvent type on tannin concentrations in the extract. The programming languages R (version 4.3.1) and RStudio (version 1.4.1717) were used to perform a series of statistical tests to properly analyze the data. The Shapiro–Wilk statistical test was used to determine the data distribution. The Analysis of Variance (ANOVA) was used to examine normally distributed data, while the Kruskal–Wallis statistical test was used to examine non-normally distributed data. In addition, the post hoc Tuckey test was used to detect statistically significant differences between concentrations in normally distributed data. Similarly, the post hoc Dunn test was used to detect statistically significant differences in non-normally distributed data.

## 4. Conclusions

In the realm of sustainable extraction practices, evaluating the energy efficiency and environmental impact of different extraction methods is crucial. Our study examined various techniques for isolating ellagitannins from wood, with a focus on their energy-saving potential and environmental friendliness. The extraction efficiency of ellagitannins, such as vescalagin and castalagin, varies significantly when different solvents, process conditions, and methodologies are applied. This research employed several extraction techniques, including supercritical CO_2_ extraction, ultrasonic extraction, cold maceration, and Soxhlet extraction, using various solvent systems such as EtOH, H_2_O, EtOH + H_2_O, Ac, and Ac + H_2_O mixtures. Each method has distinct advantages and drawbacks. Supercritical CO_2_ extraction is highly effective but requires substantial energy for pressurization and heating. It is eco-friendly due to its use of non-toxic CO_2_, which can be recycled, minimizing solvent waste. Ultrasonic extraction is energy-efficient as it operates at lower temperatures and reduces extraction time. It uses less solvent and is considered “green” due to its minimal environmental impact and energy conservation. Cold maceration is energy-saving as it operates at room temperature but requires longer extraction times, which can be a drawback despite its low environmental impact. Soxhlet extraction is energy-intensive due to continuous heating and solvent reflux, using a large amount of solvent and generating more waste, making it less environmentally friendly. Solvent systems such as ethanol and water are more environmentally friendly and require less energy compared to organic solvents like acetic acid. Mixed solvents can balance efficiency and environmental impact. Ultrasound-assisted extraction emerged as an efficient and economically viable method characterized by lower solvent consumption, reduced extraction temperatures, and minimal environmental impact. This study promotes the applicability of sustainable extraction methods in line with current trends in green processing and process intensification. In comparing the efficiency of novel and conventional methods, sustainable methods demonstrated comparable extraction efficiencies and higher selectivity towards the compounds of interest. Therefore, these methods should be promoted over conventional ones that require high processing temperatures and higher solvent consumption. Further investigation should focus on optimizing these methods to enhance their energy efficiency and environmental sustainability, as well as evaluating the feasibility of pilot and industrial-scale applications. 

Regarding the applicability of the extracts, they may serve as efficient natural anticancer and antimicrobial agents. For instance, a water-based ultrasound extract significantly reduced metabolic activity in WM-266-4 melanoma cells at 0.005 mg/mL without harming normal melanocytes, suggesting potential as a cancer therapy. Additionally, the extract showed antimicrobial activity against *Staphylococcus aureus* and *Escherichia coli* but not *Candida albicans*, indicating selective efficacy.

These findings underscore the importance of optimized extraction methods for ellagitannin recovery and highlight their potential therapeutic applications. 

## Data Availability

The original contributions presented in the study are included in the article, further inquiries can be directed to the corresponding author.

## Abbreviations

SCF40/250 + EtOH	Supercritical extraction with CO_2_ at 40 °C and 250 bar with the cosolvent ethanol
SCF40/250 + (EtOH + H_2_O)	Supercritical extraction with CO_2_ at 40 °C and 25 bar with the cosolvent ethanol + water
SCF40/250 + H_2_O	Supercritical extraction with CO_2_ at 40 °C and 250 bar with the cosolvent water
SCF40/250 + Ac	Supercritical extraction at 40 °C and 250 bar with the cosolvent acetone
SCF40/250 + (Ac + H_2_O)	Supercritical extraction with CO_2_ at 40 °C and 250 bar with the cosolvent acetone + water
UE (EtOH)	Ultrasound extraction with the solvent ethanol
UE (EtOH + H_2_O)	Ultrasound extraction with the solvent ethanol + water
UE (H_2_O)	Ultrasound extraction with the solvent water
UE (Ac)	Ultrasound extraction with the solvent acetone
UE (Ac + H_2_O)	Ultrasound extraction with the solvent acetone + water
CM (EtOH)	Cold maceration with the solvent ethanol
CM (EtOH + H_2_O)	Cold maceration with the solvent ethanol + water
CM (H_2_O)	Cold maceration with the solvent water
CM (Ac)	Cold maceration with the solvent acetone
CM (Ac + H_2_O)	Cold maceration with the solvent acetone + water
SOX (EtOH)	Soxhlet extraction with the solvent ethanol
SOX (EtOH + H_2_O)	Soxhlet extraction with the solvent ethanol + water
SOX (H_2_O)	Soxhlet extraction with the solvent water
SOX (Ac)	Soxhlet extraction with the solvent acetone
SOX (Ac + H_2_O)	Soxhlet extraction with the solvent acetone + water

## Appendix A

**Table A1:** Recovery of ellagitannins extracted using SCE (mg/g).

	SCF40/250 + EtOH	SCF40/250 + H_2_O	SCF40/250 + (EtOH + H_2_O)	SCF40/250 + Ac	SCF40/250 + (Ac + H_2_O)
**vescalin**	4.89	13.13	9.14	0.22	9.06
**castalin**	9.58	20.41	1.83	ND	7.39
**vescalagin**	14.36	40.86	21.28	0.83	81.48
**castalagin**	9.70	28.21	14.68	0.84	34.66

ND—not detected.

**Table A2:** Recovery of ellagitannins extracted using UE extraction (mg/g).

	UE (EtOH)	UE (H_2_O)	UE (EtOH + H_2_O)	UE (Ac)	UE (Ac + H_2_O)
**vescalin**	7.05	73.71	38.05	35.37	37.71
**castalin**	9.65	75.81	41.94	24.64	26.21
**vescalagin**	7.88	2.71	42.79	144.13	163.85
**castalagin**	5.73	23.06	29.42	60.32	69.47

**Table A3:** Recovery of ellagitannins extracted using CM (mg/g).

	CM (EtOH)	CM (H_2_O)	CM (EtOH + H2O)	CM (Ac)	CM (Ac + H_2_O)
**vescalin**	7.57	62.14	39.02	18.67	46.73
**castalin**	9.60	52.35	41.30	9.15	21.66
**vescalagin**	9.12	29.34	34.71	95.04	131.16
**castalagin**	6.15	17.20	23.28	45.85	58.04

**Table A4:** Recovery of ellagitannins extracted using SOX extraction (mg/g).

	SOX (EtOH)	SOX (H_2_O)	SOX (EtOH + H_2_O)	SOX (Ac)	SOX (Ac + H_2_O)
**vescalin**	3.30	36.30	22.74	17.54	28.87
**castalin**	17.50	34.40	37.72	14.88	22.79
**vescalagin**	5.50	52.41	30.49	134.30	175.35
**castalagin**	9.33	30.41	27.75	52.62	72.62
